# Leveraging Machine Learning to Assess Post-COVID-19 Glycemic Control in Diabetic Patients

**DOI:** 10.3390/ijerph23050644

**Published:** 2026-05-12

**Authors:** Marie Lluberes-Contreras, Eduardo Figueroa-Santiago, Hamid-Reza Kohan-Ghadr, Angel Ortiz-Ortega, Abiel Roche-Lima

**Affiliations:** 1 Bioinformatics and Data Science Laboratory, Department of Computer Science, University of Puerto Rico, San Juan, PR 00925, USA; 2 Department of Computer Science, University of Puerto Rico, San Juan, PR 00925, USA; 3 SysBioSolutions LLC, Portage, MI 49024, USA; 4 Medresearch LLC, Caguas, PR 00725, USA; info@oficinadrortiz.com; 5 Center for Collaborative Research in Health Disparities, Medical Sciences Campus, University of Puerto Rico, San Juan, PR 00936, USA

**Keywords:** machine learning, electronic health records, HbA1c, disease diagnosis, COVID-19, epidemic prediction, diabetes

## Abstract

**Highlights:**

**Public health relevance—How does this work relate to a public health issue?**

- Diabetes management is highly sensitive to healthcare disruptions, making the COVID-19 pandemic a critical context for evaluating long-term glycemic control.
- Understanding post-infection HbA1c trajectories addresses an important gap in assessing the broader population-level impact of COVID-19 on chronic disease outcomes.

**Public health significance—Why is this work of significance to public health?**

- In a large national cohort, most patients (71%) did not experience significant changes in HbA1c following COVID-19 infection.
- Key determinants of glycemic variability included clinical and structural factors such as BMI, insulin use, age, and socioeconomic proxies.

**Public health implications—What are the key implications or messages for practitioners, policy makers and/or researchers in public health?**

- Population-wide deterioration in glycemic control post-COVID-19 may be limited, but targeted interventions are needed for vulnerable subgroups.
- Integrating EHR data with interpretable machine learning can support surveillance and guide data-driven public health strategies for chronic disease management.

**Abstract:**

Hemoglobin A1c is a central biomarker for long-term glycemic control and a key predictor of diabetes-related complications. The COVID-19 pandemic disrupted routine healthcare delivery and introduced potential metabolic effects of SARS-CoV-2 infection, yet the long-term impact of COVID-19 on glycemic trajectories in individuals with diabetes remains unclear. In this retrospective study, we leveraged harmonized electronic health record data from the National Clinical Cohort Collaborative to evaluate changes in HbA1c before and after documented SARS-CoV-2 infection in adults with diabetes (*n* = 93,320). Patients were required to have repeated HbA1c measurements pre- and post-infection and stable exposure to key antihyperglycemic medications. A paired statistical analysis was used to identify individuals with statistically significant post-infection changes in HbA1c. We then developed and evaluated multiple supervised machine learning classifiers using an 80/20 train–test split and cross-validation to assess demographic, clinical, and structural factors associated with significant glycemic change. Most patients (71%) did not experience a statistically significant change in average HbA1c following COVID-19 infection, and among those who did, decreases were more common than increases. A random forest classifier achieved the best overall performance, and feature importance and SHAP analyses highlighted body mass index, insulin use, age, and socioeconomic proxies as key contributors. These findings suggest that while COVID-19 infection does not substantially alter long-term glycemic control for most patients with diabetes, individual-level clinical and structural factors influence post-infection glycemic variability.

## 1. Introduction

Hemoglobin A1c (HbA1c) is a key biomarker for assessing long-term glycemic control and guiding clinical management in individuals with diabetes mellitus. Persistently elevated HbA1c levels are associated with increased risk of microvascular and macrovascular complications, including retinopathy, nephropathy, neuropathy, and cardiovascular disease, highlighting its prognostic importance in diabetes care [1,2,3]. Effective diabetes management depends not only on clinical care but also on consistent access to healthcare services, medications, self-monitoring tools, and supportive resources. Structural and social determinants of health—including socioeconomic status, insurance coverage, geographic location, and systemic inequities—have long contributed to disparities in glycemic control, disproportionately affecting vulnerable and historically underserved populations [4,5,6,7].

The COVID-19 pandemic created widespread disruptions in healthcare delivery that further challenged chronic disease management. Reductions in in-person visits, interruptions in laboratory testing, and rapid shifts to telehealth altered routine diabetes care, often with unequal adoption and effectiveness across populations [8]. In addition, SARS-CoV-2 infection itself has been associated with metabolic dysregulation through inflammatory responses, stress-related hyperglycemia, corticosteroid exposure, and potential direct effects on pancreatic β-cell function [9]. While prior studies have examined acute outcomes, incident diabetes, and dysglycemia following COVID-19 infection in patients with diabetes, including associations between pre-infection HbA1c and acute severity, hospitalization, and mortality [10], much of this work focuses on short-term prognostic endpoints rather than long-term metabolic trajectories. Longitudinal changes in HbA1c following SARS-CoV-2 infection remain poorly understood, as systematic reviews show insufficient evidence characterizing post-infection glycemic trajectories [11]. A large multicenter retrospective cohort study using National Clinical Cohort Collaborative data found no significant difference in longer-term average HbA1c following SARS-CoV-2 infection among individuals with type 2 diabetes compared with matched controls, underscoring uncertainty regarding post-infection glycemic change [12]. Additionally, prospective follow-up studies of COVID-19 survivors with dysglycemia have observed modest increases in HbA1c over time [13], suggesting potential longer-term metabolic effects. Taken together, existing evidence remains limited and inconsistent, and it remains unclear which demographic, clinical, and healthcare system-level characteristics are associated with significant post-COVID changes in glycemic control.

Machine learning methods have been widely applied to electronic health record (EHR) data for clinical risk prediction and outcome modeling. Prior studies have demonstrated the effectiveness of ensemble and deep learning approaches in capturing complex, nonlinear relationships in high-dimensional clinical datasets, often outperforming traditional statistical models [14,15]. At the same time, methodological work has highlighted challenges related to data heterogeneity, missingness, and generalizability in EHR-based modeling [16,17].

In the context of COVID-19, large-scale EHR resources such as the National Clinical Cohort Collaborative (N3C) have enabled extensive investigation of disease outcomes across diverse populations. Recent studies using N3C data have applied machine learning approaches to predict outcomes such as long COVID, hospitalization, and disease severity [18]. However, most of this work has focused on acute outcomes, with comparatively limited attention to longitudinal metabolic trajectories and post-infection glycemic control.

To address these gaps and examine these changes at scale, we leveraged harmonized EHR data from the N3C COVID Enclave, a secure centralized platform that aggregates federated data from healthcare systems across the United States [19]. In this retrospective study, we compared HbA1c measurements before and after documented SARS-CoV-2 infection and identified individuals with statistically significant changes in glycemic control. We then applied a machine learning–based classification pipeline to evaluate associations of demographic, clinical, and structural characteristics captured in de-identified EHR data with the likelihood of experiencing a significant post-COVID change in HbA1c. Importantly, this study distinguishes between statistical significance and clinical relevance, emphasizing the need to interpret observed HbA1c changes within a broader clinical context.

In this study, we pursued three complementary objectives: (1) a large-scale longitudinal characterization of HbA1c changes before and after SARS-CoV-2 infection using harmonized EHR data; (2) a patient-level statistical framework for defining significant glycemic change as a predictive outcome; and (3) an interpretable machine learning analysis identifying clinical and structural determinants associated with post-infection glycemic variability.

## 2. Materials and Methods

Throughout this manuscript, we use the term variables to refer to raw clinical data elements extracted from the EHR, and features to refer to processed or derived representations of these variables used as inputs to machine learning models.

All computations were performed in the N3C integrated closed platform. We conducted our analysis and implemented our models using Python 3.9 and PySpark 3.5.

### 2.1. Data Source and Cohort Creation

This retrospective study used harmonized electronic health data from the N3C COVID Enclave. The enclave aggregates federated data from participating healthcare systems across the United States and standardizes disparate clinical data into a common data model while preserving site-level provenance, providing a longitudinal, demographically diverse cohort.

We identified adults (≥18 years) with a documented positive SARS-CoV-2 test and a diagnosis of diabetes mellitus. To reduce confounding related to treatment changes, medication exposure was controlled for insulin, glucagon-like peptide-1 (GLP-1) receptor agonists, and metformin. Medication exposure was defined using structured EHR indicators derived from recorded medication orders and prescription records within the N3C data model. Binary indicators were created to reflect whether patients had documented exposure to each medication class during the pre- and post-COVID periods. Continuous exposure was defined as consistent presence (or absence) of these indicators across both periods. Individuals were required to have consistent medication status for each class—either continuously exposed both before and after SARS-CoV-2 infection or unexposed during both periods. These drug classes have been shown to be associated with different clinical outcomes in patients with diabetes and COVID-19, and failure to account for their use can introduce confounding by indication in observational analyses [20].

To ensure reliable assessment of longitudinal glycemic change, patients were required to have at least three HbA1c measurements before infection and at least three measurements after infection. Longitudinal glycemic studies typically require repeated measurements to characterize within-individual change, and prior observational research has included individuals with three or more HbA1c measurements to define temporal trends [21,22]. Patients with insufficient HbA1c measurements or with missing or unclear values in key analytic variables, BMI and Diabetes diagnostic indicator, were excluded from the final analytic cohort.

HbA1c measurements derived from EHR data are irregularly sampled and reflect differences in healthcare utilization patterns across patients. Despite this, they provide a valuable real-world representation of longitudinal glycemic control in routine clinical practice. To characterize the temporal distribution of HbA1c measurements, we defined the observation window for each individual as the time between the first and last recorded HbA1c measurement within each period. We also computed the interval between consecutive measurements. The median observation window was 1000 days (IQR: 686–1352) pre-infection and 748 days (IQR: 484–1013) post-infection. The median interval between measurements was similar across periods (140 [IQR: 103–189] vs. 127 days [IQR: 98–182]), suggesting comparable measurement frequency. Pre- and post-infection HbA1c values were defined relative to the date of confirmed SARS-CoV-2 infection, with measurements aggregated within their respective observation periods.

**Figure 1:**
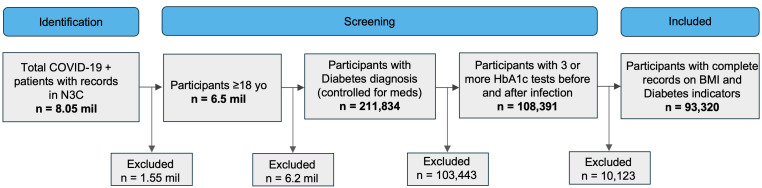
Derivation of the study cohort from the N3C COVID Enclave. Patients were sequentially filtered based on COVID-19 test positivity, age, diabetes diagnosis, medication exposure criteria, availability of pre- and post-infection HbA1c measurements, and data completeness.

**Figure 2:**
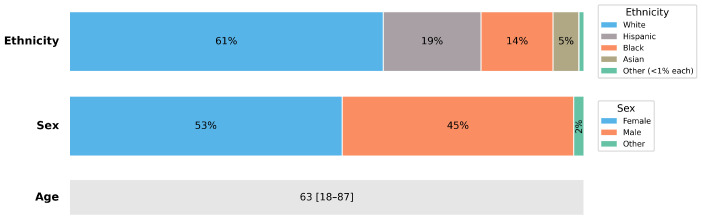
Demographic characteristics of the study cohort. Ethnicity and sex are displayed as stacked horizontal bars representing the proportion of participants in each category. Age is summarized as the median with minimum and maximum values. Ethnicity categories with individual representation below 1% were grouped into a single category (“Other (<1% each)”) for visualization clarity. These categories include Native American, Pacific Islander and Other non-Hispanic.

From an initial population of approximately 8.05 million COVID-19–positive patients in the N3C Enclave, 6.5 million adults were identified. Of these, 211,834 had a diagnosis of diabetes with controlled medication exposure, and 108,391 met the requirement of having at least three HbA1c measurements before and after infection. After applying data completeness criteria, the final analytic cohort consisted of 93,320 patients. Figure 1 illustrates the cohort selection and analytic sample derivation. Baseline cohort demographics are summarized in Figure 2.

### 2.2. Outcome Definition, Feature Engineering, and Statistical Analysis

The primary outcome was defined as a binary indicator of a statistically significant change in HbA1c following documented SARS-CoV-2 infection. The significance of HbA1c change for each patient was determined using paired *t*-tests comparing pre- and post-infection measurements. We selected paired *t*-tests to capture within-individual changes in HbA1c while accounting for repeated measurements before and after infection. This approach provides a consistent patient-level assessment of change across individuals with varying measurement frequency and follow-up duration, while maintaining interpretability and suitability as a classification target. Exploratory analysis of within-person HbA1c differences indicated an approximately symmetric distribution with mild skewness (−0.39), supporting the use of paired *t*-tests. Given the large sample size, the analysis is robust to moderate deviations from normality.

Prior to model training, we performed feature preprocessing to reduce redundancy and multicollinearity, and improve model stability. Features with low variance—defined as having the same value in 95% or more of observations—were removed. Pairwise Pearson correlation coefficients were computed to identify highly correlated features; when two features had a correlation ≥ 0.8, one feature was removed. The 95% threshold for low-variance filtering was selected to remove features with minimal discriminatory power, a common practice in high-dimensional clinical datasets. The Pearson correlation threshold of 0.8 was chosen to reduce multicollinearity while preserving clinically meaningful variables, consistent with standard feature selection practices in applied machine learning [23].

**Figure 3:**
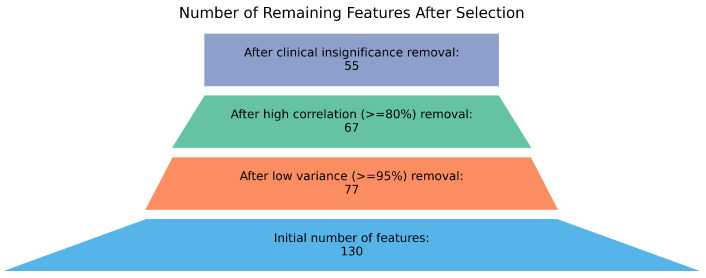
Feature selection process. From bottom: Original data set consist of 130 features, of which 53 showed near-zero variance. After correlation analysis, 10 of the remaining features had correlation coefficients ≥ 0.8. Twelve additional features deemed not clinically meaningful were removed.

Features deemed clinically meaningful were retained, even if they met low variance or high-correlation criteria. Clinical feature selection was guided by domain knowledge, prioritizing variables with known relevance to glycemic control and cardiometabolic risk (e.g., BMI, insulin use, comorbidities). This hybrid approach was chosen to balance statistical redundancy reduction with clinical interpretability, which is critical in EHR-based modeling. This feature reduction process is illustrated in Figure 3. Records with missing values in these selected features were excluded prior to model fitting. All preprocessing steps were performed independently of the outcome to prevent data leakage. A full list of all candidate variables and their corresponding feature-selection decisions, including those retained or removed based on clinical evaluation, is provided in Table A1, Appendix A.

### 2.3. Machine Learning Model Development and Evaluation

#### 2.3.1. Candidate Algorithms and Initial Model Fitting

We evaluated multiple supervised classification algorithms representing linear, nonlinear, tree-based, instance-based, and probabilistic modeling approaches. Specifically, we considered linear discriminant analysis (LDA), logistic regression (LR), support vector machine (SVM), k-nearest neighbors (KNN), decision tree classifier (classification and regression tree; CART), random forest classifier (RFC), gradient boosting classifier (GBC), and Gaussian naïve Bayes (NB).

LDA and LR are linear classifiers that model class separation through linear combinations of input features, with LDA assuming multivariate normality and shared covariance across classes [24,25]. SVM constructs a maximum-margin decision boundary and can capture nonlinear relationships through kernel functions [26]. KNN is an instance-based method that classifies observations based on the labels of their nearest neighbors in feature space [27].

Tree-based methods included CART, which recursively partitions the feature space using decision rules [28], and RFC, an ensemble method that aggregates predictions from multiple decision trees trained on bootstrapped samples with random feature selection [29]. Gradient boosting builds an ensemble of weak learners sequentially, where each model corrects errors made by prior models [30]. Gaussian naïve Bayes is a probabilistic classifier that assumes conditional independence among features and models class-conditional feature distributions as Gaussian [31].

Prior to model training, the dataset was randomly split into 80% training and 20% held-out testing sets. All experiments were conducted using a fixed random seed to ensure reproducibility. Cross-validation was applied during model training using k-fold splits (k = 10). Given the large sample size, class proportions remained stable across folds, ensuring consistent performance estimates. All models were trained and evaluated using two approaches: the first approach used raw data, while the second approach used scaled data, to account for differences in scale sensitivity across algorithms [32]. Feature scaling parameters were estimated using the training data only using the scikit-learn library. Model performance was assessed using cross-validation within the training set to identify candidate algorithms for further optimization. To address class imbalance, we applied random oversampling to the minority class on the training data only, using the RandomOverSampler method from the imbalanced-learn library. We selected random oversampling due to its simplicity and its ability to balance class distributions by duplicating existing minority class observations without introducing synthetic samples, which is desirable in high-dimensional EHR data. Model performance was evaluated using metrics robust to class imbalance, including precision, recall, and their harmonic mean, the F1-score [33]. Final model performance was reported on the held-out test set.

#### 2.3.2. Feature Selection Using Permutation Feature Importance

Permutation feature importance was computed on the best-performing model to identify the top 15 features contributing most to predictive performance. These features were then evaluated from a clinical perspective to ensure relevance and interpretability, and used for hyperparameter tuning and final model fitting. All calculations were restricted to training data to avoid leakage.

#### 2.3.3. Hyperparameter Tuning and Final Model Fitting

Hyperparameter optimization was performed within training folds. The final model was then fitted on the full training set and evaluated on a held-out test set.

#### 2.3.4. Post Hoc Model Interpretation

After fitting the final model, SHAP (Shapley Additive Explanations) values were computed to quantify the contribution of each predictor to individual predictions and overall model output. SHAP analysis was applied post hoc to the final model for interpretability.

**Figure 4:**
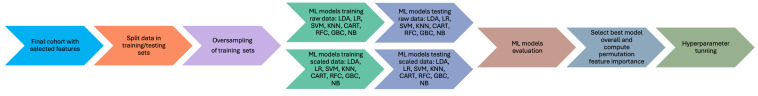
Machine Learning pipeline. After data preparation, cohort creation and feature selection, data is split in 80% for training and 20% for testing. Then, random oversampling is applied to the training data. The 8 models are trained with raw and scaled data. After testing, the feature permutation importance is computed for the best performing model. Finally, parameters are tuned for the selected model.

Figure 4 illustrates our Machine Learning model development and evaluation pipeline.

## 3. Results

We present our results following the order of our analysis.

### 3.1. Statistical Analysis

**Figure 5:**
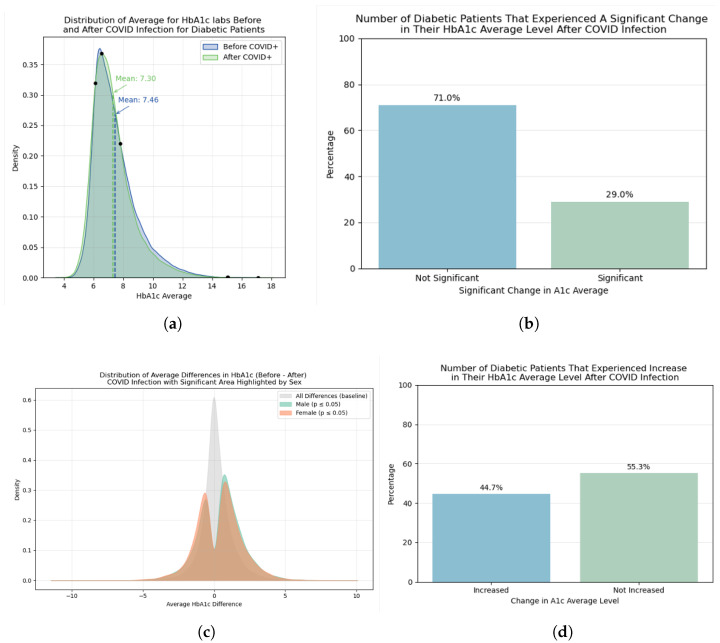
Some results from statistical distribution of HbA1c levels before and after COVID-19 positive test and paired *t*-test significance test. (**a**) Distribution of average values of HbA1c before and after COVID-19 positive test; (**b**) Percentage of patients with significant difference in HbA1c average values before and after COVID-19 positive test; (**c**) Distribution by sex of difference in HbA1c values before and after COVID-19 positive test; (**d**) Percentage of patients with and without increased average HbA1c levels post COVID-19 positive test.

Figure 5a shows the distribution of the average of HbA1c laboratory tests for all patients before and after COVID-19 infection, with means for each distribution annotated on the graph. Both distributions show a similar pattern. Both pre- and post-COVID HbA1c distributions were right-skewed, with slightly lower median values observed in the post-infection period.

The paired *t*-test showed that for 71.0% of the patients, the difference between their average HbA1c values before and after infection was not significant (*p*-value > 0.05), as shown in Figure 5b. We also computed this distribution for patients with incomplete health records, showing a 75.0% of patients without significant change. While statistical significance was assessed using paired tests, the magnitude of HbA1c changes should be interpreted in context, as small differences may reach statistical significance in large samples without necessarily reflecting clinically meaningful changes.

The distribution by sex of the differences between the labs averages used for the *t*-test is shown in Figure 5c. Positive differences were more frequent than negative differences for both sexes, indicating that pre-infection HbA1c values were generally higher. Figure 5d shows that 44.7% of the patients with significant change in HbA1c values experienced an increase in their average results, vs. 55.3% experienced no increase (i.e., stable or decreased HbA1c). This proportion is shared by the set of patients that included incomplete records, with 44.96% and 55.04% for increase and not increase among significant changes respectively, showing consistency on the distribution across the cohort creation.

### 3.2. Model Evaluation

The mean scores for accuracy, precision, recall, F1 and ROC_AUC for the models described in Section 2.3.1 are shown in Table 1, with the unscaled RFC achieving the best results for accuracy and F1, and second best for precision. Tree-based models (RFC and CART) consistently outperformed linear and instance-based methods, suggesting that nonlinear relationships and feature interactions play an important role in predicting glycemic change. In contrast, linear models such as logistic regression and LDA showed lower performance, indicating that the relationship between predictors and outcome is not well captured by linear decision boundaries. These results are consistent with the ability of ensemble methods to capture nonlinear relationships in clinical data. The scaled RFC achieved a slightly better score for ROC_AUC, while both scaled and unscaled CART models achieved better recall, with RFC as second in general. Although in diagnostics, recall scores—also called sensitivity or True Positive Rate—are usually prioritized, our target predicts a significant change in value without caring for the direction of the change. This means the value may have increased or decreased. Precision, also called Positive Predictive Value, can tell us how accurately the model captures these changes. We favored the selection of RFC to model this behavior in part because it shows the higher F1-score. F1 is the harmonic mean of precision and recall, balancing both. Model performance metrics are reported as mean and standard deviation across cross-validation folds. For the selected random forest model, performance variability was low, indicating stable predictive behavior. The random forest model was further evaluated after hyperparameter tuning (n_estimators = 98) using 5-fold cross-validation (490 total fits), and final performance was reported on the held-out test set.

To further assess classification behavior, we examined model performance across classes using precision and recall metrics. The results indicate balanced performance between the two classes, with comparable precision and recall values. The slightly higher precision relative to recall for the positive class suggests that the model is somewhat conservative in predicting significant HbA1c change, favoring fewer false positives at the expense of some false negatives. Misclassification is likely concentrated among patients with smaller or borderline differences in HbA1c values, where distinguishing statistically significant from non-significant change is challenging due to measurement variability and clinical heterogeneity. Class-level performance metrics for the final model are summarized in Table 2.

### 3.3. Permutation Feature Importance

**Figure 6:**
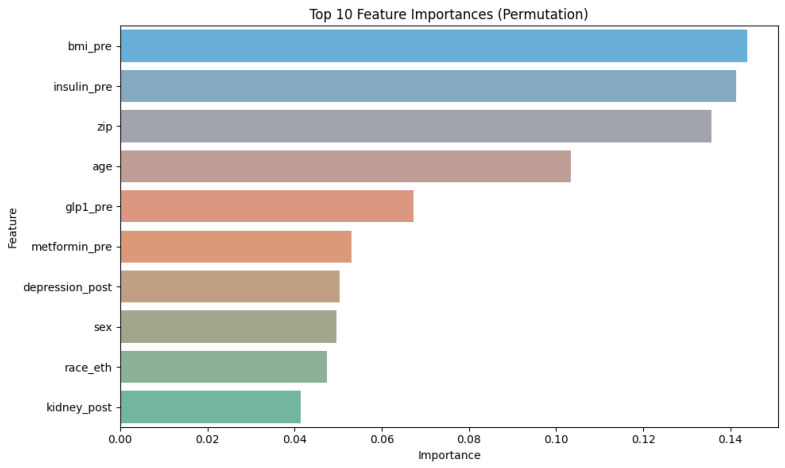
Top 10 features and their importance in the models. Features labeled “-pre” refer to conditions that occurred previous COVID-19 infection, and those labeled “-pro” occurred after infection.

Figure 6 shows the top features and their ranking value, in decreasing order, with body mass index (BMI), insulin intake previous COVID-19 infection and zip code appearing as the 3 more important features. Age and other glucose control medications follows. Permutation Feature Importance helps to understand which features are more important for the model stability by measuring the impact each feature has on the model performance. We discuss the 15 most import features in the next section. A full list of all features and their importance is shown in Appendix A, Table A2.

### 3.4. SHAP Values

**Figure 7:**
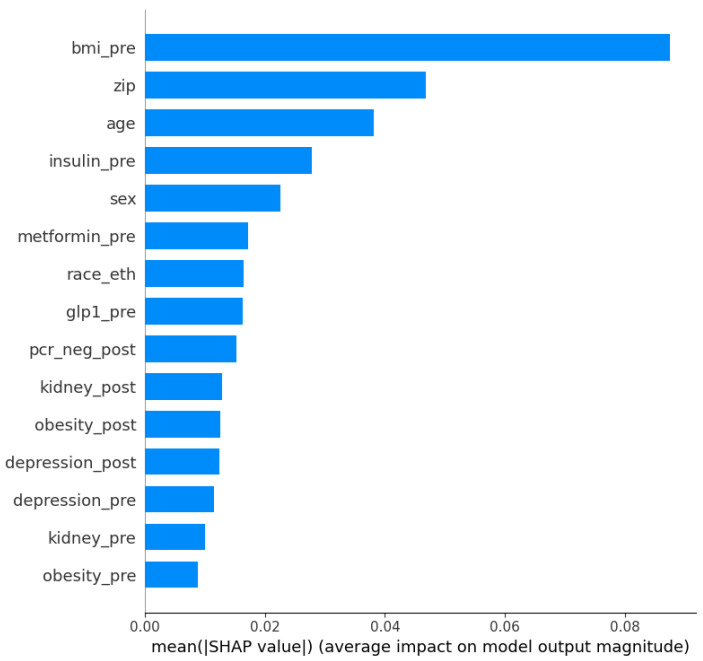
Impact of individual features in the model. Features labeled “-pre” refer to conditions that occurred previous COVID-19 infection, and those labeled “-pro” occurred after infection.

Shapley Additive Explanations, or SHAP, measure the magnitude of individual feature contributions to the model. To ensure interpretability at the cohort level, we focused on global explanation methods, which capture overall feature contributions across the population. Figure 7 shows the top 15 features. Although SHAP and Feature Permutation importance use different mechanisms to quantify features’ impact on the models, the results obtained here are very similar for both analysis.

## 4. Discussion

### 4.1. Summary of Findings

In this retrospective N3C cohort of 93,320 adults with diabetes, most patients (71%) did not experience a statistically significant change in average HbA1c following documented SARS-CoV-2 infection. Among the 29% with a statistically significant within-patient change, decreases were more common than increases (55.3% versus 44.7%). At the cohort level, mean HbA1c shifted modestly from 7.46% pre-infection to 7.30% post-infection, and both pre- and post-infection distributions remained right-skewed with a reduction in the upper tail after infection. These findings suggest that, for most individuals with diabetes, SARS-CoV-2 infection was not associated with substantial long-term changes in glycemic control, although a subset of patients exhibited variability. This overall stability is consistent with prior studies reporting limited long-term impact of COVID-19 on HbA1c at the population level, while also highlighting heterogeneity across individuals.

Among the eight candidate models, the random forest classifier achieved the best overall performance on the held-out test set (accuracy 0.93, F1 0.93, precision 0.97, recall 0.90, AUROC 0.95). This likely reflects both the ability of ensemble methods to capture nonlinear relationships in EHR data and structural aspects of the outcome definition. Because the outcome is based on within-individual statistical comparisons of HbA1c measurements, features related to baseline glycemic control and measurement patterns may contribute to class separability in addition to underlying clinical differences. These considerations are important when interpreting model performance in observational EHR-based studies.

Permutation feature importance and SHAP analyses converged on a core set of metabolic, demographic, and contextual variables that consistently contributed to model predictions of post-infection glycemic change: BMI, insulin exposure, ZIP code, age, GLP-1 and metformin exposure, post-infection depression, sex, race/ethnicity, and post-infection kidney disease.

### 4.2. Statistical vs. Clinical Significance

The primary outcome of this study was defined statistically, via a paired *t*-test on within-patient HbA1c measurements, and this should be distinguished from clinical significance. HbA1c reflects long-term glycemic control and is associated with risk of diabetes-related complications; however, small changes may reach statistical significance in large samples without substantially altering clinical management. Current guidance and minimum clinically important difference estimates place a meaningful HbA1c change at approximately 0.3–0.5% points absolute, the range typically used to guide treatment intensification or to declare therapeutic efficacy in clinical trials [34]. The population mean difference observed here, 0.16% points, falls below this threshold. Even within the subgroup with a statistically significant within-patient change, many differences likely reflect within-individual laboratory variability and biological noise rather than a shift that would prompt a change in therapy. The interpretation of the 29% “significant change” group are the patients with detectable within-person HbA1c movement across repeated measurements, not necessarily as a group with clinically actionable deterioration or improvement. A magnitude-based outcome, for example an absolute within-patient change of at least 0.5 percentage points, is a natural extension for future analyses and is likely to yield a smaller but more clinically interpretable positive class.

### 4.3. Interpretation of Predictive Features

The following paragraphs summarize key predictors identified by the model and provides clinical context for their association with glycemic variability. These interpretations describe plausible associations and should not be interpreted as causal relationships. Because the model is trained on structured EHR features that reflect both underlying biology and the healthcare processes that generate the data, each feature should be read as a marker that carries predictive information in this dataset, not as a lever that, if modified, would change post-COVID HbA1c.

#### 4.3.1. Cardiometabolic Burden

BMI and the obesity diagnosis code, together with insulin, metformin, and GLP-1 receptor agonist exposure, dominated the ranking. Within this cohort these features are most consistent with markers of baseline dysglycemia severity and treatment intensity rather than independent drivers of post-infection change. Adiposity is well established as a correlate of insulin resistance and chronic low-grade inflammation [35,36]; GLP-1 exposure in structured EHR data is largely a marker of GLP-1 receptor agonist prescription in higher-risk metabolic patients [37]; and metformin exposure tracks diagnosed type 2 diabetes or prediabetes.

#### 4.3.2. Demographic and Structural Context

Age, sex, race/ethnicity, and postal code contributed substantially. Older age is associated with higher baseline glycemic risk in this cohort [38]. ZIP code carries area-level information correlated with social determinants of health, including neighborhood socioeconomic status, food environment, care continuity, and chronic stress exposure [39]. However, the use of area-level proxies (e.g., postal code) may not fully capture individual-level socioeconomic conditions and may introduce ecological bias. Sex and race/ethnicity likely capture a combination of biological variation, structural differences in access and diagnosis, and site-level documentation patterns within N3C [40].

#### 4.3.3. Comorbid Burden and Treatment Exposure

Kidney disease, chronic lung disease, hypertension, depression, and systemic corticosteroid exposure (both pre- and post-infection) also ranked prominently. Systemic corticosteroids are of particular interest because they are simultaneously a marker of acute disease severity and a known perturbant of glucose homeostasis [41]; in this observational setting the two roles cannot be separated. Kidney disease [42], hypertension [43], and depression [44] are all recognized correlates of dysglycemia in the general diabetes literature and plausibly function here as additional markers of overall cardiometabolic and psychosocial burden.

#### 4.3.4. Post-Infection Documentation Markers

Post-COVID PCR negativity and post-infection depression, kidney, and obesity codes also ranked highly. These features likely reflect continued healthcare engagement and documentation intensity after the index infection rather than de novo pathology, and they must be interpreted with that caveat. They are informative for the model because patients who remain in active follow-up are both the patients on whom HbA1c can be remeasured and the patients on whom new comorbidities are most likely to be documented.

Overall, the top predictors are consistent with a profile of patients at higher baseline cardiometabolic risk and with greater healthcare engagement, and with the well-known association of corticosteroid exposure with short-term dysglycemia. The present analysis is associational and does not establish that SARS-CoV-2 infection, as opposed to the pandemic-era clinical pathways that surrounded it, is responsible for the observed patterns.

### 4.4. Limitations

Given the retrospective, EHR-based nature of this study, several considerations are important for contextualizing the results. First, the use of observational data limits causal interpretation, as observed associations may reflect a combination of infection effects, healthcare disruptions, and underlying patient characteristics. Second, although internal validation supports model robustness within this dataset, generalizability to other populations remains to be established. Third, HbA1c measurements are irregularly sampled and influenced by healthcare utilization patterns, which may affect estimation of glycemic trends. Fourth, structured EHR variables may not fully capture all relevant behavioral, social, or treatment-related factors, and residual confounding may persist despite efforts to account for medication exposure.

These findings should also be interpreted in light of healthcare utilization patterns, as EHR-derived measurements reflect differences in follow-up intensity and access to care. Patients with more frequent monitoring may be more likely to exhibit detectable changes, independent of underlying physiological differences, from patients who disengaged from care during the pandemic. The “no significant change” group may in particular be enriched for patients with stable, well-monitored diabetes.

In addition, ZIP code is an area-level measure and does not capture within-area heterogeneity in individual socioeconomic position; inferences made at the individual level from an area-level proxy are therefore subject to ecological bias. ZIP code is also susceptible to misclassifications: residential moves may not be captured consistently in the EHR, a single ZIP code may aggregate structurally different neighborhoods, and what a given ZIP code actually proxies (access to preventive care, food environment, air quality, chronic stress exposure) varies across regions. The feature’s high ranking in our model should therefore be read as evidence that area-level context carries predictive signal, not as a precise estimate of any one socioeconomic mechanism.

Finally, results should be considered within the context of the U.S. healthcare system, and may not fully generalize to settings with different healthcare structures, access patterns, or diabetes management practices.

Taken together, these constraints reinforce that our findings describe associations within a real-world U.S. EHR cohort and should not be read as causal estimates of SARS-CoV-2 effects on glycemic trajectories. These considerations highlight opportunities for future work to further strengthen and extend the present findings.

## 5. Conclusions

In this large retrospective analysis of EHR data from the N3C COVID Enclave, we found that the majority of adults with diabetes did not experience statistically significant changes in average HbA1c following SARS-CoV-2 infection. Among patients who did exhibit significant changes, reductions in HbA1c were more frequent than increases, suggesting that post-COVID glycemic deterioration is not universal and may be influenced by contextual factors beyond infection alone.

Our machine learning framework identified body mass index, insulin exposure, age, and socioeconomic proxies such as postal code as key predictors of post-infection glycemic change, underscoring the importance of both biological and structural determinants of health. The strong performance within this dataset of the random forest model, combined with interpretable feature importance and SHAP analyses, supports the utility of explainable machine learning approaches for studying heterogeneous outcomes in large, real-world clinical datasets.

These results contribute to a growing body of evidence suggesting that long-term glycemic control after COVID-19 is relatively stable for most individuals with diabetes, while highlighting subgroups that may be more vulnerable to post-infection metabolic changes. These findings highlight the importance of distinguishing statistically significant changes from clinically meaningful ones and support the use of predictive models as tools for risk stratification and monitoring rather than definitive clinical decision-making. Future work should explore integrating both statistical and clinical criteria, incorporate longer follow-up periods, more granular measures of healthcare utilization, and causal modeling approaches to better disentangle the effects of infection, treatment disruptions, and social determinants on glycemic trajectories. Further methodological extensions, including ablation-style analyses and external or temporal validation, may complement the interpretability analyses presented in this study by more systematically assessing feature contributions and model transportability.

## Figures and Tables

**Table 1:** Mean scores for models in the pipeline. SC stands for scaled for models using scaled data. StD is the metric standard deviation.

Model	Accuracy	F1	Precision	Recall	ROC_AUC	StD Acc	StD F1	StD Prc	StD Rec	StD ROC
LDA	0.5806	0.5373	0.5991	0.4872	0.6173	0.0061	0.0074	0.0083	0.0086	0.0063
LDA SC	0.5806	0.5373	0.5991	0.4872	0.6173	0.0061	0.0074	0.0083	0.0086	0.0063
GDB	0.6022	0.4584	0.718	0.3368	0.647	0.0031	0.0045	0.0174	0.0033	0.0072
GDB SC	0.6022	0.4584	0.718	0.3368	0.647	0.0031	0.0045	0.0174	0.0033	0.0072
LR	0.5777	0.5422	0.5920	0.5001	0.6155	0.0065	0.0072	0.0085	0.0079	0.0064
LR SC	0.5777	0.5422	0.5920	0.5001	0.6155	0.0065	0.0072	0.0085	0.0079	0.0064
NB	0.5577	0.5727	0.5592	0.6087	0.6023	0.0087	0.0534	0.0307	0.1187	0.0071
NB SC	0.5214	0.6334	0.5200	0.8586	0.6023	0.0215	0.0557	0.0294	0.1891	0.0071
RFC	0.9337	0.9313	0.9672	0.8979	0.9505	0.0022	0.0028	0.0042	0.0036	0.0024
RFC SC	0.9334	0.9309	0.9666	0.8978	0.9511	0.0021	0.0028	0.0037	0.0034	0.0026
KNN	0.6936	0.6912	0.6967	0.6859	0.7700	0.0042	0.0055	0.0087	0.0046	0.0047
KNN SC	0.6643	0.6811	0.6487	0.7170	0.7332	0.0031	0.0044	0.0063	0.0036	0.0032
SVM	0.5901	0.3119	0.9704	0.1858	0.6232	0.0043	0.0057	0.0114	0.0039	0.0065
SVM SC	0.6637	0.6054	0.7322	0.5161	0.7224	0.0046	0.0085	0.0104	0.0081	0.0064
CART	0.8239	0.8400	0.7697	0.9244	0.824	0.0033	0.0041	0.0061	0.0046	0.0025
CART SC	0.8233	0.8395	0.7688	0.9247	0.8234	0.0036	0.0042	0.0060	0.0042	0.0029

**Table 2:** Classification performance of the final random forest model on the test set. 0 and 1 are the predicted classes, where 0 means the difference in HbA1c levels was not significant and 1 that it was.

	Precision	Recall	F1-Score	Support
0	0.8866	0.9747	0.9285	9338.0
1	0.9723	0.8768	0.9221	9276.0
accuracy			0.9254	18,664.0
macro avg	0.9294	0.9257	0.9253	18,664.0
weighted avg	0.9297	0.9254	0.9253	18,664.0

**Figure A1:**
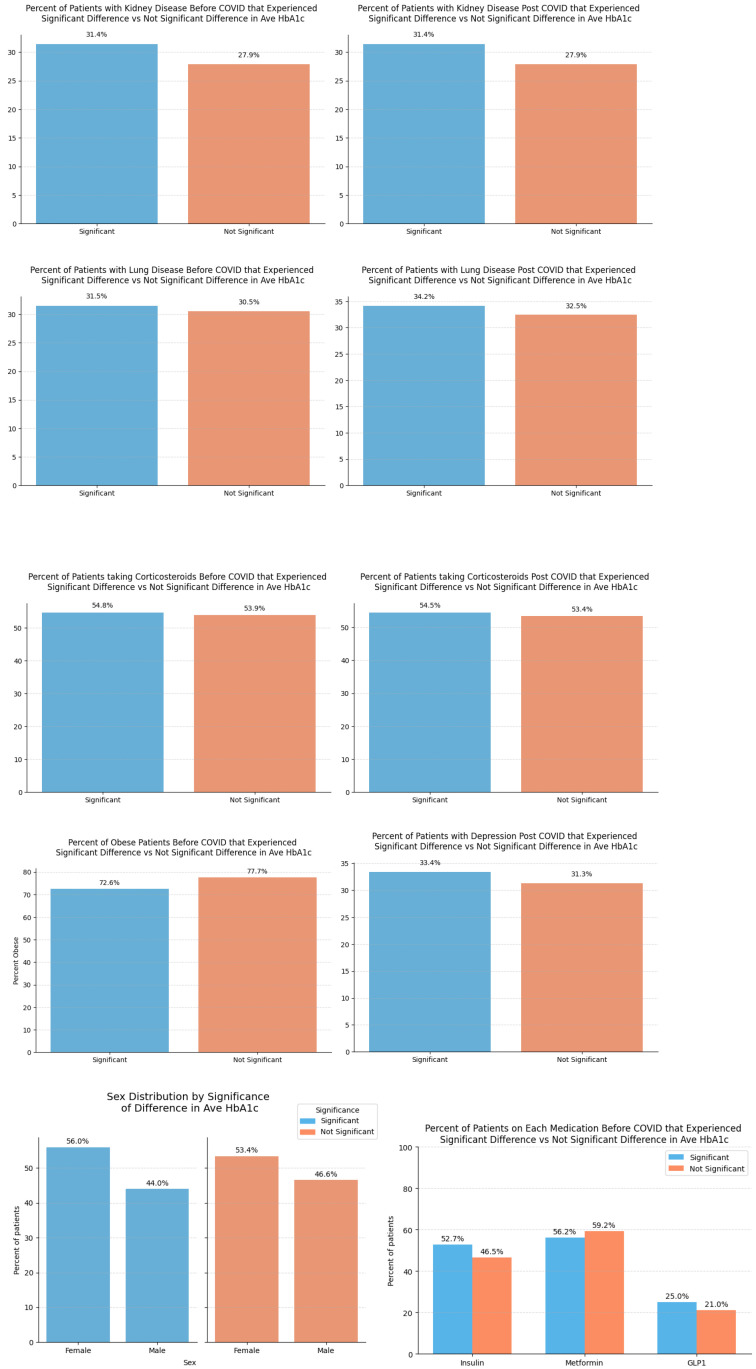
Percentage of patients with and without significant HbA1c differences for most important features.

**Figure A2:**
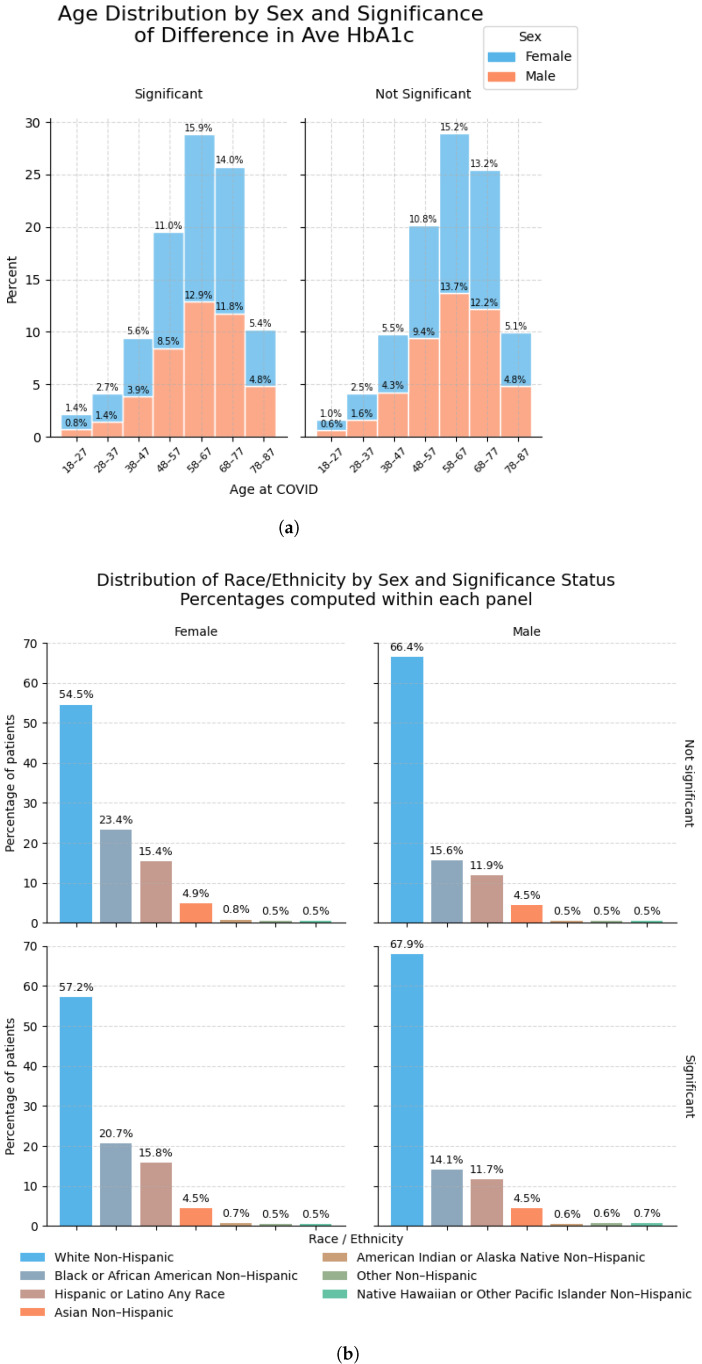
Patients distribution per sex and by (**a**) age and (**b**) ethnicity, by significance in HbA1c difference before and post COVID infection.

## Data Availability

The data used for this research is federated data in the centralized, secure platform N3C. The data version for the cohort used for this project is ri.foundry.main.build.0f061a69-ace6-4028-a88e-75bea10e30b2.

## Abbreviations

The following abbreviations are used in this manuscript:

HbA1c	Hemoglobin A1c
EHR	Electronic health records
N3C	National Clinical Cohort Collaborative
GLP-1	Glucagon-like peptide-1
LDA	Linear discriminant analysis
LR	Logistic regression
SVM	Support vector machine
KNN	k-nearest neighbors
CART	Classification and regression tree
GBC	Gradient boosting classifier
NB	Gaussian naïve Bayes
SHAP	Shapley Additive Explanations

## Appendix A

**Table A1:** All features importance.

Feature	Included	Excluded
sex	✓	
postal code	✓	
age at covid	✓	
race		correlated to race ethnicity
race ethnicity	✓	
COVID first poslab or diagnosis date		not clinically relevant
number of visits before covid		not clinically relevant
number of visits post covid		not clinically relevant
BMI max observed or calculated before or day of covid	✓	
BMI max observed or calculated post covid		correlated to BMI before covid
DIABETESCOMPLICATED before or day of covid indicator		COHORT
DIABETESCOMPLICATED post covid indicator		COHORT
DIABETESUNCOMPLICATED before or day of covid indicator		low variance
DIABETESUNCOMPLICATED post covid indicator		low variance
Insulin before or day of covid indicator	✓	
Insulin during weak covid hospitalization indicator		correlated to covid weak
Insulin during strong covid hospitalization indicator		correlated to covid string
Insulin post covid indicator		correlated to insulin before covid
Metformin before or day of covid indicator	✓	
Metformin during weak covid hospitalization indicator		low variance
Metformin during strong covid hospitalization indicator		low variance
Metformin post covid indicator		correlated to Metformin before covid
GLP1 before or day of covid indicator	✓	
GLP1 during weak covid hospitalization indicator		low variance
GLP1 during strong covid hospitalization indicator		low variance
GLP1 post covid indicator		correlated to GLP1 before covid
TUBERCULOSIS before or day of covid indicator		low variance
MILDLIVERDISEASE before or day of covid indicator	✓	
MODERATESEVERELIVERDISEASE before or day of covid indicator		low variance
THALASSEMIA before or day of covid indicator		low variance
RHEUMATOLOGICDISEASE before or day of covid indicator	✓	
DEMENTIA before or day of covid indicator		low variance
CONGESTIVEHEARTFAILURE before or day of covid indicator		correlated to Heart failure before covid
SUBSTANCEUSEDISORDER before or day of covid indicator	✓	
DOWNSYNDROME before or day of covid indicator		low variance
KIDNEYDISEASE before or day of covid indicator	✓	
MALIGNANTCANCER before or day of covid indicator	✓	
CEREBROVASCULARDISEASE before or day of covid indicator	✓	
PERIPHERALVASCULARDISEASE before or day of covid indicator	✓	
PREGNANCY before or day of covid indicator		low variance
HEARTFAILURE before or day of covid indicator	✓	
HEMIPLEGIAORPARAPLEGIA before or day of covid indicator		low variance
PSYCHOSIS before or day of covid indicator		low variance
OBESITY before or day of covid indicator	✓	
CORONARYARTERYDISEASE before or day of covid indicator	✓	
SYSTEMICCORTICOSTEROIDS before or day of covid indicator	✓	
DEPRESSION before or day of covid indicator	✓	
METASTATICSOLIDTUMORCANCERS before or day of covid indicator		low variance
HIVINFECTION before or day of covid indicator		low variance
CHRONICLUNGDISEASE before or day of covid indicator	✓	
PEPTICULCER before or day of covid indicator		low variance
SICKLECELLDISEASE before or day of covid indicator		low variance
MYOCARDIALINFARCTION before or day of covid indicator	✓	
CARDIOMYOPATHIES before or day of covid indicator	✓	
HYPERTENSION before or day of covid indicator	✓	
OTHERIMMUNOCOMPROMISED before or day of covid indicator	✓	
Antibody Neg before or day of covid indicator		low variance
PULMONARYEMBOLISM before or day of covid indicator		low variance
TOBACCOSMOKER before or day of covid indicator	✓	
SOLIDORGANORBLOODSTEMCELLTRANSPLANT before or day of covid indicator		
Antibody Pos before or day of covid indicator		low variance
SUBSTANCEABUSE before or day of covid indicator		low variance
number of COVID vaccine doses before or day of covid		low variance
LL IMV during strong covid hospitalization indicator		low variance
COVID patient death during strong covid hospitalization indicator		low variance
COVIDREGIMENCORTICOSTEROIDS during strong covid hospitalization indicator		
COVID diagnosis during strong covid hospitalization indicator	✓	
REMDISIVIR during strong covid hospitalization indicator		low variance
LL ECMO during strong covid hospitalization indicator		low variance
LL IMV during weak covid hospitalization indicator		low variance
COVID patient death during weak covid hospitalization indicator		low variance
COVIDREGIMENCORTICOSTEROIDS during weak covid hospitalization indicator	✓	
COVID diagnosis during weak covid hospitalization indicator	✓	
REMDISIVIR during weak covid hospitalization indicator		low variance
LL ECMO during weak covid hospitalization indicator		low variance
TUBERCULOSIS post covid indicator		low variance
PCR AG Pos post covid indicator	✓	
MILDLIVERDISEASE post covid indicator	✓	
MODERATESEVERELIVERDISEASE post covid indicator		low variance
PNEUMONIADUETOCOVID post covid indicator	✓	
THALASSEMIA post covid indicator		low variance
RHEUMATOLOGICDISEASE post covid indicator	✓	
DEMENTIA post covid indicator		low variance
CONGESTIVEHEARTFAILURE post covid indicator		correlated to heart failure post covid
SUBSTANCEUSEDISORDER post covid indicator	✓	
Long COVID clinic visit post covid indicator		low variance
DOWNSYNDROME post covid indicator		low variance
KIDNEYDISEASE post covid indicator	✓	
MALIGNANTCANCER post covid indicator	✓	
MISC post covid indicator		not clinically relevat
CEREBROVASCULARDISEASE post covid indicator	✓	
PERIPHERALVASCULARDISEASE post covid indicator	✓	
PREGNANCY post covid indicator		low variance
HEARTFAILURE post covid indicator	✓	
HEMIPLEGIAORPARAPLEGIA post covid indicator		low variance
PSYCHOSIS post covid indicator		low variance
OBESITY post covid indicator	✓	
CORONARYARTERYDISEASE post covid indicator	✓	
PCR AG Neg post covid indicator	✓	
SYSTEMICCORTICOSTEROIDS post covid indicator	✓	
DEPRESSION post covid indicator	✓	
METASTATICSOLIDTUMORCANCERS post covid indicator		low variance
HIVINFECTION post covid indicator		low variance
CHRONICLUNGDISEASE post covid indicator	✓	
B94 8 post covid indicator		low variance
PEPTICULCER post covid indicator		low variance
SICKLECELLDISEASE post covid indicator		low variance
MYOCARDIALINFARCTION post covid indicator	✓	
Long COVID diagnosis post covid indicator		low variance
CARDIOMYOPATHIES post covid indicator	✓	
HYPERTENSION post covid indicator	✓	
OTHERIMMUNOCOMPROMISED post covid indicator	✓	
Antibody Neg post covid indicator		low variance
PULMONARYEMBOLISM post covid indicator		low variance
TOBACCOSMOKER post covid indicator	✓	
SOLIDORGANORBLOODSTEMCELLTRANSPLANT post covid indicator		correlated to organ blood before covid
Antibody Pos post covid indicator		low variance
SUBSTANCEABUSE post covid indicator		low variance
number of COVID vaccine doses post covid		low variance
had at least one reinfection post covid indicator	✓	
first strong COVID ED only start date		not clinically relevant
first strong COVID hospitalization start date		not clinically relevant
first strong COVID hospitalization end date		not clinically relevant
first weak COVID ED only start date		not clinically relevant
first weak COVID hospitalization start date		not clinically relevant
first weak COVID hospitalization end date		not clinically relevant
strong COVID hospitalization length of stay	✓	
COVID patient death indicator		low variance
death within specified window post covid		low variance
Severity Type	✓	

**Table A2:** All features importance and their importance for the RFC. “pre” means the condition was present pre-COVID-19 positive test and “post” after positive test.

Feature	Importance
bmi pre	0.141396
Insulin pre	0.141396
zip	0.135734
age	0.103332
glp1 pre	0.067215
metformin pre	0.053020
depression post	0.050375
sex	0.049614
race eth	0.047418
kidney post	0.041369
pcr neg post	0.033666
steroids post	0.030580
obesity pre	0.028747
depression pre	0.024772
systemic corticost pre	0.021803
kidney disease pre	0.020540
obesity post	0.020406
chronic lung dis pre	0.020175
chronic lung dis post	0.017139
severity type	0.015279
hyperten pre	0.015082
hyperten post	0.014113
smoker post	0.011404
pcr pos post	0.011185
cancer post	0.010510
coronary dis post	0.010433
heart fai post	0.010323
p vascular dis post	0.010069
liver post	0.009950
smoker pre	0.009240
p vascular dis pre	0.008828
liver dis pre	0.008625
immunocompr post	0.008321
subst use pre	0.007943
rheum post	0.007509
coronary dis pre	0.007491
rheum pre	0.007020
malcancer pre	0.006849
subst use post	0.006723
myo inf post	0.006625
immunocompr pre	0.005865
reinfection post	0.005562
stroke post	0.005338
stroke pre	0.004279
heart fai pre	0.004213
covid strong	0.003632
myo inf pre	0.002588
pneumonia post	0.002144
cardiomyopathy post	0.002107
covid dx weak	0.001835
covid dx strong	0.001408
cardiomyopathy pre	0.001397
transplant pre	0.001211
covidsteroids weak	0.001170
covidsteroids strong	0.000800
